# Pennsylvania State Veterinary Medical Association

**Published:** 1887-04

**Authors:** 


### PROCEEDINGS VETERINARY MEDICAL SOCIETIES.

### PENNSYLVANIA STATE VETERINARY MEDICAL ASSOCIATION.

Annual meeting held in Philadelphia, March 14th, 1887, at the Veterinary Department of the University of Pennsylvania. President J. W. Sallade in the chair.

The following officers were elected for the ensuing year: President, Thomas B. Rayner; first vice president, W. L. Zuill; second vice president, W. H. Knight; third vice president, P. M. Minster; corresponding secretary, Alexander Glass; recording secretary, Robert Gladfelter; treasurer, J. C. Fly; trustees, J. C. Michener, W. H. Hoskins, T. B. Rayner, W. S. Kooker, R. S. Huidekoper.

Dr. Hoskins then presented the following draft of a bill to regulate the practice of veterinary medicine in the Commonwealth, which received the approval of the association:

AN ACT TO REGULATE THE PRACTICE OF VETERINARY MEDICINE AND SURGERY IN PENNSYLVANIA.

SECTION I. *Be it enacted by the Senate and House of Representatives of the Commonwealth of Pennsylvania, in General Assembly met, and it is hereby enacted by the authority of the same,* That every person who shall assume, or use, or cause to be used, any title pertaining to the practice of Veterinary Medicine or Surgery, or any of the branches of Veterinary Medicine or Surgery, shall be a graduate of a legally-chartered Veterinary College or University, having the power or authority to confer the degree of Veterinary Surgeon or analogous title; except as provided for in SECTION II. And such practitioner shall be required to register in the book kept for that purpose, in the Office of the Prothonotary of the County in which he resides.

SEC. II. Any person who has assumed the title of Veterinary Surgeon or analogous title, in this Commonwealth, for the five years preceding the passage of this Act, without being entitled to the degree of Veterinary Surgeon or analogous title, shall be allowed to continue the use of the title; but such person shall appear before the Prothonotary of the County in which he resides and make affidavit of that fact; he shall then be recorded as an "existing practitioner."

SEC. III. The Prothonotary shall purchase a book of suitable size, to be known as the "Veterinary Medical Register" of the County, and shall set apart one full page for the registration of each practitioner; and, when any practitioner shall die or remove from the County, the Prothonotary shall make a note of the same, and shall perform such other duties as are required by this Act.

SEC. IV. Every practitioner who shall be admitted to register shall pay to the Prothonotary the sum of one dollar; which sum shall be compensation in full for registration. The Prothonotary shall give a receipt for the same, and such registration shall take place within six months from the passage of this Act.

SEC. V. Nothing in this Act shall be so construed as to prevent any Veterinary Surgeon (if legally qualified to use the title) from using the title of "Veterinary Surgeon" or analogous title in this Commonwealth; but if such Veterinary Surgeon opens an office or uses the title for the transaction of business, he shall be deemed a "sojourner" and shall conform to the requirements of this Act.

SEC. VI. Any person who may desire to commence the practice of Veterinary Surgery or Medicine, or any of its branches, in this State, after the passage of this Act, and who holds a Veterinary diploma, issued, or purporting to have been issued, by any Veterinary College or University in this State, another State or foreign country, shall make affidavit before the Prothonotary that his diploma has been regularly issued by a legally-chartered Veterinary College or University, after which such person will be allowed to register as provided for in this Act.

SEC. VII. Any person who shall present to a Prothonotary a Veterinary diploma which has been obtained fraudulently; or which is, in whole or in part, a forgery; or shall make affidavit to any false statement, intended to be filed or registered; or shall use the title of Veterinary Surgeon or analogous title, without conforming to the requirements of this Act; or shall otherwise violate or neglect to comply with any of the provisions of this Act, shall be deemed guilty of a misdemeanor, and, on conviction, shall be punished for each and every offence, by a fine of one hundred dollars—one-half to be paid to the prosecutor and the other half to be paid to the County; or shall be imprisoned in the County jail of the proper County for a term not exceeding one year, or both or either, at the discretion of the Court.

A committee on legislation was appointed, consisting of Drs. W. H. Hoskins, W. S. Kooker, W. L. Zuill, R. S. Huidekoper, C. C. McLean, M. Recktenwald, James B. Rayner.

Dr. Hoskins read a paper on Azoturia, which called forth, from Drs. Michener, Zuill, Recktenwald, Hart and Huidekoper, a short discussion on the pathology of this disease, which showed that our knowledge of its exact pathology is yet in an unsatisfactory condition.

Dr. Glass presented a paper on the use of lanoline as a base for ointments, and claimed for it unusual properties of absorption.

Dr. Huidekoper presented a complete set of lesions of farcy glanders from a case which had been brought to the hospital, asking for treatment, after having been in the hands of a quack of the town, and under treatment for six months. Attention was urgently called to the necessity of proper legislation upon this subject in Pennsylvania, as the Commonwealth is at present entirely destitute of any laws which provide for the suppression of the disease or for the destruction of the animals affected.

Dr. McLean presented the outline of a bill now before the State Legislature, which provides that all stallions which stand for service in the

State shall pay a license fee of fifty dollars and be registered ; shall not be registered without a certificate of soundness (from hereditary vices) from a qualified veterinarian ; also, that the owner of said horse shall have a lien on mare and foal for service money during a period of nine months after foaling. After some short discussion, this bill received the approval of the association. The approval was also given to a bill now before the State Legislature which appropriates $100,000 to the Veterinary Department of the University of Pennsylvania for the completion of its buildings and laboratories.

A petition was signed by the officers and all members present.

Adjournment until the semi-annual meeting in Pittsburg, the second Tuesday of September, 1887.

---

THE UNITED STATES VETERINARY MEDICAL ASSOCIATION

